# The Croatian Primary Sjögren’s Disease Oral Health Study: Oral Status and Oral Health-Related Quality of Life

**DOI:** 10.3390/jcm12144804

**Published:** 2023-07-21

**Authors:** Ana Glavina, Ivona Božić, Katica Parat, Dijana Perković, Dolores Biočina-Lukenda, Dušanka Martinović Kaliterna, Mislav Radić

**Affiliations:** 1 Dental Clinic Split, 21000 Split, Croatia; glavina2014@gmail.com (A.G.); katicaparat@gmail.com (K.P.); dolores.biocina-lukenda@mefst.hr (D.B.-L.); 2 Department of Oral Medicine and Periodontology, Study of Dental Medicine, School of Medicine, University of Split, 21000 Split, Croatia; 3 Division of Rheumatology and Clinical Immunology, Center of Excellence for Systemic Sclerosis in Croatia, University Hospital Split, 21000 Split, Croatia; ivona.bozic7@gmail.com (I.B.); dijana.perkovic@hotmail.com (D.P.); martikalit@gmail.com (D.M.K.); 4 School of Medicine, University of Split, 21000 Split, Croatia

**Keywords:** dental caries, oral health, periodontal diseases, quality of life, saliva, Sjögren’s syndrome

## Abstract

To determine salivary flow rate, oral and periodontal status, oral health-related quality of life (OHRQoL), objective and subjective indexes, and serum antibody reactivity in patients with primary Sjögren’s disease (pSD). Thirty-one patients with pSD and 31 control subjects participated in this cross-sectional, single-center study. The unstimulated whole salivary flow rate (UWSFR) and stimulated whole salivary flow rate (SWSFR), salivary pH, DMFT index (DMFT = D—decayed, M—missing, F—filled tooth), periodontal pocket depth (PPD), clinical attachment level (CAL), interincisal distance, OHRQoL, objective European League Against Rheumatism (EULAR) SS Disease Activity Index (ESSDAI) and subjective (EULAR SS Patient Reported Index (ESSPRI), 6-items-VAS-SS (Visual Analog Scale), Profile of Fatigue) indexes were analyzed. The patients with pSD had a blood sample taken in the morning between 7 and 10 a.m. for comprehensive laboratory analysis. Patients with pSD had statistically significant lower UWSFR (0.20 vs. 0.90 mL/min) and SWSFR (0.56 vs. 1.64 mL/min) values compared with control subjects (*p* < 0.001, Mann-Withney U test). Salivary pH value of pSD patients was significantly lower compared with control subjects (6.00 vs. 7.00; *p* < 0.001, Mann-Whitney U test). The mean DMFT index of patients with pSD compared to control subjects was not statistically significant (23.74 ± 7.28 vs. 20.77 ± 5.73; *p* = 0.08, *t*-test). Interincisal distance was significantly decreased in the pSD group compared with control subjects (43.80 ± 0.38 vs. 47.60 ± 0.50; *p* = 0.003, *t*-test). The prevalence of periodontitis was similar in patients with pSD and control subjects (83.9% vs. 77.4%; *p* = 0.35, λ^2^ test). The mean Oral Health Impact Profile (OHIP-49) total score was statistically significantly higher in pSD patients compared with control subjects (32.00 vs. 8.00; *p* < 0.001, Mann–Whitney U test). Patients with pSD have decreased salivary flow and salivary pH, poor oral health, decreased interincisal distance, high prevalence of periodontitis, and worse OHRQoL. These findings highlight the need for a multidisciplinary approach to the management of patients with pSD that includes physical and psychological aspects of the disease.

## 1. Introduction

Hyposalivation, with or without xerostomia, is one of the earliest and most common oral symptoms in patients with primary Sjögren’s disease (pSD). Primary Sjögren’s disease (SD) is a chronic lymphoproliferative systemic autoimmune disease characterized by the destruction of the lacrimal and salivary glands [1]. The disease is associated with a significantly increased risk of non-Hodgkin’s lymphoma (NHL) of the B-cell series, occurring in approximately 5.0% of patients [2]. In addition, dryness may affect other mucosal surfaces such as the respiratory tract, digestive tract, and vagina, leading to the clinical picture of “sicca syndrome” or “sicca complex” [3]. As a result, hyposalivation leads to a series of oral cavity difficulties: difficulty in speaking, chewing, swallowing, wearing mobile prosthetic dentures, glossitis, glossodynia, dysgeusia, oral candidiasis, cheilitis angularis, accelerated development of dental caries, acute infections of the major salivary glands, traumatic oral lesions. The relationship between SD and periodontitis has not been proven. The studies performed are very heterogeneous, so a comparison is not possible. The results of two systematic reviews and meta-analyzes comparing periodontal parameters of patients with SD with control subjects differed [4].

A recent study showed that health-related quality of life (HRQoL) scores of patients with pSD were comparable to those observed in other autoimmune diseases generally considered more aggressive (i.e., systemic lupus erythematosus (SLE), rheumatoid arthritis (RA)) [5]. HRQoL is a group of parameters introduced in dentistry to evaluate the quality of life (QoL) of patients. Some of the first studies on HRQoL were related to third molar extraction, and later the use of these parameters spread throughout dentistry [6,7]. The concept of QoL in the context of oral health was developed relatively late, in the early 1980s [8]. Several instruments have been developed to measure the impact of oral health on HRQoL. The Oral Health Impact Profile (OHIP) is one of the most sophisticated and widely used instruments [9]. In the study by Lόpez-Jornet P. et al., the Spanish validated version of the OHIP-49 was used to assess oral HRQoL (OHRQoL). Scores for all items of the OHIP-49 were lower in patients with pSD compared with control subjects [10].

The European League Against Rheumatism (EULAR) initiated the development of standardized instruments to assess objective and subjective parameters for pSD. First, the EULAR Sjögren’s Syndrome Disease Activity Index (ESSDAI) was defined in 2009—the index of disease activity (objective) [11]. Two years later, another standardized instrument was defined to assess subjective patient complaints, i.e., the EULAR Sjögren’s Syndrome Patient Reported Index (ESSPRI). It represents the middle of three existing scales: Patient Global Assessment (PGA), Sicca Symptoms Inventory (SSI), and Profile of Fatigue and Discomfort (PROFAD) [12]. These two instruments are well-defined and validated, and their use is recommended in both clinical trials and clinical practice. Recently, new classification criteria and disease activity scores have been developed, which are primarily intended for research purposes but may also be useful in daily clinical practice [3].

The objectives of our study are to determine salivary flow rate [unstimulated whole salivary flow rate (UWSFR), stimulated whole salivary flow rate (SWSFR)]; oral status [salivary pH, interincisal distance, DMFT index (DMFT = D—decayed, M—missing, F—filled tooth)]; prevalence of periodontitis; OHRQoL; objective (ESSDAI) and subjective (ESSPRI, 6-items-VAS-SS (Visual Analog Scale), Profile of Fatigue) indexes; and serum antibody reactivity in patients with pSD.

## 2. Materials and Methods

### 2.1. Study Design and Subjects

This was a single-center, cross-sectional study. The cross-sectional study was conducted from July 2018 to March 2020 at the Department of Rheumatology and Clinical Immunology, Center of Excellence for Systemic Sclerosis in Croatia, University Hospital Split, Split, Croatia, and at the Dental Clinic (Teaching Base of the School of Medicine, Study of Dental Medicine, University of Split, Split, Croatia). The study was approved by the Ethics Committee of the School of Medicine, University of Split, Split, Croatia, on 12 May 2016 (class: 003-08/16-03/0001; registration number: 2181-198-03-04-16-0022) and was conducted in accordance with the principles of the 1964 Declaration of Helsinki and its subsequent amendments. All subjects were enrolled after explanation of the study protocol and signing of the informed consent form. Thirty-one patients with pSD and 31 control subjects participated in the cross-sectional study. All subjects were ≥18 years of age. Patients with pSD were matched for age and sex with control subjects for comparison.

The diagnosis of pSD was based on the 2016 American College of Rheumatology/European League Against Rheumatism (ACR/EULAR) classification criteria [13]. The co-author of this study (M.R.) performed all rheumatologic examinations of the pSD patients. An experienced periodontist and co-author (K.P.) performed all initial periodontal examinations, including sialometric assessment, salivary pH, DMFT index, interincisal distance, and OHRQoL.

The control group consisted of patients with degenerative joint disease. Minor salivary gland biopsy (MSGB) was not performed in the control group, following the guidelines of the local Ethics Committee.

The exclusion criteria were:

- Intake of anticholinergics, tricyclic antidepressants, antihypertensives, and antihistamines; systemic diseases (such as anorexia, bulimia, diabetes, HIV infection, hepatitis C (HCV)) that could cause salivary gland dysfunction,
- Radiation therapy to the head and neck region,
- Smoking.

### 2.2. Outcomes Measures

Three sets of outcomes were obtained: 1. salivary flow rate (UWSFR, SWSFR), salivary pH, interincisal distance, DMFT index, periodontal status, OHRQoL, 2. ESSDAI, ESSPRI, 6-items-VAS-SS, Profile of Fatigue, 3. serum antibody reactivity. UWSFR and SWSFR were collected in graduated tubes with an opening of 1.50 cm diameter using the “spit method”. Sialometry was performed following the guidelines of Navazesh [14]. The pH of saliva was determined using indicator paper, which was left in the oral cavity for one minute. The color of the indicator paper was compared with the color of the attached scale (Merck KGaA, Darmstadt, Germany). Saliva was considered acidic or alkaline depending on whether the salivary pH was below or above the reference value (the salivary pH reference value is 6.00 to 6.50). The interincisal distance was measured at the beginning of the periodontal examination to avoid bias caused by prolonged opening of the mouth. Patients were asked to open their mouth as much as possible. The interincisal distance was defined as the distance between the incisal edge of the lower central incisor and the incisal edge of the upper central incisor [15]. Interincisal distance was measured in edentulous subjects with dentures. Dental status was assessed using the DMFT index, excluding third molars [16]. If carious teeth were suspected, radiographic examination was performed in addition to the clinical examination. The initial periodontal examination was performed according to a standardized protocol using a dental mirror and a CPI (Community Periodontal Index) probe in daylight [17]. The applied probe force was equivalent to the weight of 25.00 g (0.025 kg × 9.81 m/s^2^). Data were recorded in the form recommended by the World Health Organization [18]. The index values of CPI were as follows: 0—healthy periodontium, 1—gingival bleeding after probing, 2—calculus and bleeding, 3—shallow periodontal pockets (4.00–5.00 mm), and 4—deep periodontal pockets (6.00 mm and more). Periodontal pocket depth (PPD) was measured twice on all teeth. If the values obtained differed by >2.00 mm, the measurement was performed a third time and the two closest values were recorded. The PPD values at each site (mesio-buccal, mid-buccal, disto-buccal, mesio-lingual, mid-lingual, disto-lingual) were averaged. Clinical attachment level (CAL) was measured once for each tooth. The presence of periodontitis on a given tooth was defined as either PPD > 3.00 mm or CAL ≥ 5.50 mm [19,20]. The Croatian version of the OHIP-49 was used to assess OHRQoL, with no changes from the published version [21].

An objective, standardized ESSDAI instrument was used to assess disease activity in pSD patients. The ESSDAI is a clinical index consisting of 12 organ-specific domains representing each possible complication of pSD. Disease activity was categorized into 3 or 4 levels (0, 1, 2, 3) for each domain, ranging from 0 to a maximum of 3 (2) depending on severity. The ESSDAI maximum score of 27.00 indicates the highest level of disease activity [11]. The ESSPRI is a simple index used to measure the subjective complaints of pSD patients. The ESSPRI scale consists of three individual scales ranging from 0–10, i.e., VAS for each domain (dryness, pain, somatic fatigue). The maximum score is 30.00, with a higher score reflecting more severe difficulties [12]. We also used a modified self-assessment scale of the patient’s subjective complaints with 6-items-VAS-SS. This is a modified extended ESSPRI scale. It consists of six individual scales ranging from 0–10 cm, i.e., VAS for each domain (dryness, arthralgia, myalgia, paresthesia, somatic fatigue, mental fatigue) (Appendix A). The maximum total score is 60.00, with a higher score reflecting more severe difficulties. The one-dimensional Profile of Fatigue scale (VAS) is a modified scale ranging from 0—asymptomatic to 10—severe symptoms (pain, fatigue, dryness, paresthesia, myalgia and arthralgia) to assess patients’ fatigue. We used this scale to assess subjective complaints such as dryness and pain [22].

The patients with pSD had a blood sample taken in the morning between 7 and 10 a.m. for comprehensive laboratory analysis, following the hospital laboratory guidelines. The comprehensive laboratory analysis included a complete blood count (CBC) with differential, erythrocyte sedimentation rate (ESR), C-reactive protein (CRP), antinuclear antibodies (ANA), and extractable nuclear antigens (ENA). The analyzes were performed at the Department of Medical Laboratory Diagnostics, Clinical Hospital Center Split, Split, Croatia. Titers of anti-Scl-70, antiSSA/Ro60, anti-La/SSB, anti-Ro52/TRIM21 were determined by enzyme-linked immunosorbent assay (ELISA) (Cat. No. BI-5000; Biomedica, Vienna, Austria) according to the manufacturer’s instructions. Anti-centromere antibodies (ACA) were determined by indirect immunofluorescence assay (IFA) on HEp-2 cells.

### 2.3. Statistical Analysis

All analyzes were performed using IBM SPSS Statistics, version 22.00 (IBM Corp., Armonk, NY, USA). The distribution of continuous variables in the groups was expressed as mean ± standard deviation. When the distribution deviated from normal, the median was used as the mean, and the interquartile range (IQR) was used as the dispersion indicator. Normality was tested using the Kolmogorov–Smirnov test. The binomial test was used to determine if the observed categorical variable was present in a proportion that was different from 50.0%. If the *p*-value < 0.05, the presence of the selected variable cannot be determined. The *t*-test was used for differences in numerically normally distributed values, whereas the Mann–Whitney U test was used in the case of distribution deviation. Associations between qualitative variables were analyzed with the chi-square (λ^2^) test, the λ^2^ test for linear trends, or Fisher’s exact test, as appropriate. Linear correlations between continuous variables were assessed using Spearman’s rank coefficient. *p*-values < 0.05 were considered statistically significant in all data analyzes.

## 3. Results

### 3.1. Study Subjects

A total of 31 patients with pSD and 31 control subjects were included in the cross-sectional study. Most patients with pSD were women (90.0% women; mean age 51.25 (45.00–67.00)). The mean age of the control subjects was 52.25 (46.00–65.00) years, and most of them were also women. There were no significant differences between the groups with regards to age and gender. A comparison of the observed variables between pSD patients and control subjects is shown in Table 1 and Table 2.

### 3.2. Oral Status and OHRQoL

**Figure 1:**
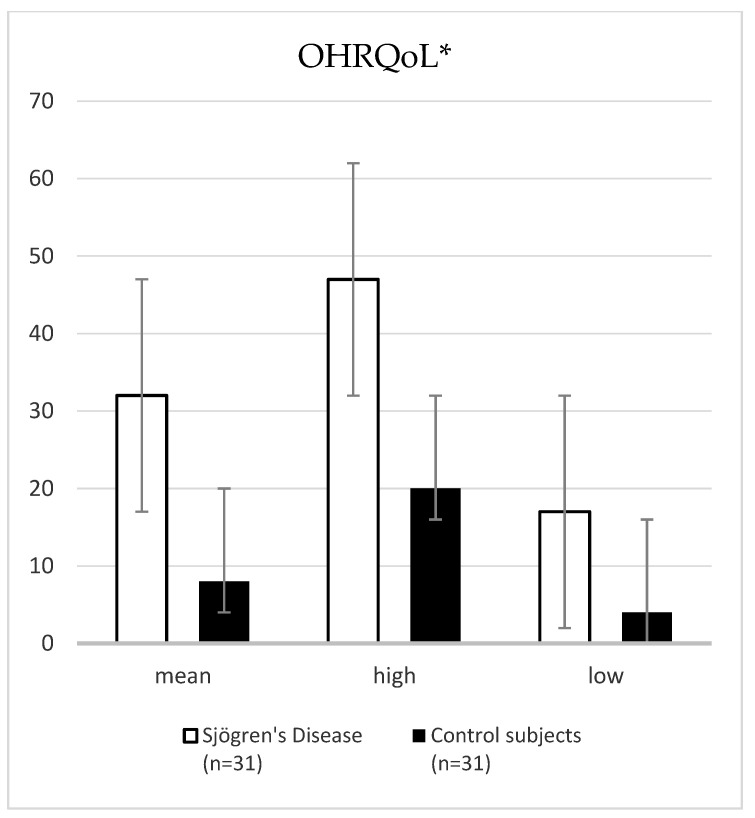
Comparison of the median and range values of the total OHIP-49 scores between patients with pSD and control subjects. * Z-4.12, *p* < 0.001. Abbreviation: OHRQoL, oral health-related quality of life.

Primary SD patients had statistically significantly lower UWSFR (0.20 vs. 0.90 mL/min) and SWSFR (0.56 vs. 1.64 mL/min) values compared with control subjects (*p* < 0.001, Mann–Withney U test). Age was not a confounding factor for sialometric assessment between groups. Salivary pH of patients with pSD was significantly lower compared to control subjects (6.00 vs. 7.00; *p* < 0.001, Mann–Whitney U test). The mean DMFT index of patients with pSD compared to control subjects was not statistically significant (23.74 ± 7.28 vs. 20.77 ± 5.73; *p* = 0.08, *t*-test). The interincisal distance significantly decreased in the pSD group compared to control subjects (43.80 ± 0.38 vs. 47.60 ± 0.50; *p* = 0.003, *t*-test). The prevalence of periodontitis was similar in patients with pSD and control subjects (83.9% vs. 77.4%; *p* = 0.35, λ^2^ test). The mean OHIP-49 total score was statistically significantly higher in pSD patients compared to control subjects (32.00 vs. 8.00; *p* < 0.001, Mann–Whitney U test) (Figure 1).

### 3.3. ESSDAI, ESSPRI, 6-Items-VAS-SS, Profile of Fatigue

The mean scores of the ESSDAI, the ESSPRI, 6-items-VAS-SS, and Profile of Fatigue are shown in Table 1. The ESSDAI correlated positively with the UWSFR, SWSFR, and OHIP-49 total score (*p* < 0.001, r = 0.708; *p* < 0.001, r = 0.743; *p* < 0.001, r = 0.949; Spearman’s rank coefficient, respectively). The ESSDAI correlated negatively with salivary pH (*p* = 0.02, r = −0.406). The ESSPRI correlated positively with the UWSFR, SWSFR, and total OHIP-49 score (*p* < 0.001, r = 0.650; *p* < 0.001, r = 0.703; *p* < 0.001, r = 0.887, respectively). The ESSPRI showed a negative correlation with salivary pH (*p* = 0.01, r = −0.441). The 6-items-VAS-SS correlated positively with the OHIP-49 total score (*p* < 0.001, r = 0.936) and negatively with UWSFR, SWSFR, and salivary pH (*p* < 0.001, r = −0.599; *p* < 0.001, r = −0.664; *p* = 0.004, r = −0.501, respectively). The Profile of Fatigue correlated positively with the OHIP-49 total score (*p* < 0.001, r = 0.978). There was a negative correlation of the Profile of Fatigue with UWSFR, SWSFR, and salivary pH (*p* < 0.001, r = −0.630; *p* < 0.001, r = −0.700; *p* = 0.02, r = −0.413, respectively). The correlation of the observed variables with the ESSDAI, ESSPRI, 6-items-VAS-SS, and Profile of Fatigue is shown in Table 3.

### 3.4. Serum Antibody Reactivity

ANA was most common in pSD patients (100.0%), followed by anti-La/SSB (64.5%) and anti-Ro/SSA and anti-TRIM21, which were equally common (61.3%). Primary SD patients with anti-Ro/SSA and anti-TRIM21 antibodies in comparison to pSD patients without them had significantly lower values of UWSFR (0.04 vs. 0.62, *p* < 0.001) and SWSFR (0.28 vs. 0.94, *p* < 0.001), lower salivary pH values (5.50 vs. 6.50, *p* = 0.03), and higher values of total OHIP-49 score (40.00 vs. 14.00, *p* < 0.001; Fisher’s exact test). Furthermore, pSD patients with anti-La/SSB antibodies had statistically significant lower values of UWSFR (0.04 vs. 0.60, *p* = 0.001) and SWSFR (0.30 vs. 0.96, *p* < 0.001) and higher values of total OHIP-49 score (39.00 vs. 14.00, *p* < 0.001; Fisher’s exact test) compared to pSD patients without these antibodies. There was no statistically significant difference in UWSFR, SWSFR, and salivary pH values (Mann–Whitney U test) between pSD patients with ribonucleoprotein (RNP) autoantibodies and pSD patients without these antibodies. There was no correlation between periodontitis and the observed autoantibodies (anti-Ro/SSA, anti-La/SSB, anti-TRIM21, RNP) (*p* = 0.63, *p* = 0.53, *p* = 0.47, and *p* = 0.64, respectively; Fisher’s exact test).

## 4. Discussion

Our cross-sectional study showed poor oral health, a high prevalence of periodontitis, and worse OHRQoL in patients with pSD. UWSFR and SWSFR values were also significantly reduced in patients with pSD. These results are consistent with the results of a study by Xin et al. which also showed significantly lower values for UWSFR (0.20 vs. 0.50, *p* < 0.001) and SWSFR (0.40 vs. 1.10, *p* < 0.001) in patients with pSD compared to control subjects [23]. The salivary pH values in our cross-sectional study were within the reference range, but it should be noted that they are closer to the acidic medium. The study by Xin et al. is not consistent with our results. It shows significantly higher salivary pH values in patients with pSD compared to control subjects, i.e., alkaline salivary values and better buffering capacity (7.20 vs. 7.60, *p* < 0.001) [23]. These results may be explained by better oral hygiene habits, which were not included in our cross-sectional study. A systematic review by Maarse et al. and studies by Christensen et al. and Márton et al. showed statistically significant higher values of the DMFT index in patients with pSD compared with control subjects (26.20 vs. 22.10, *p* < 0.001; 27.10 vs. 23.00, *p* < 0.05, respectively) [24,25,26]. The results of these studies are consistent with the results of our cross-sectional study, however, statistical significance was not reached in our study. This may be due to the small sample size in our cross-sectional study. Primary SD patients had worse oral status due to reduced values of UWSFR, SWSFR, acidic salivary pH values, creating a medium for cariogenic bacteria on the tooth surface, i.e., the formation of caries in physiologically clean sites. The interincisal distance was significantly decreased in patients with pSD compared with control subjects. This result can be explained by hyposalivation leading to severe oral dryness, i.e., reduced mouth opening due to discomfort in the oral cavity, resulting in difficulty in performing dental procedures and traumatic and infectious oral lesions (cheilitis angularis). The impact of impaired pSD immune response on periodontitis and the relationship between pSD and periodontitis remain unknown. A nationwide population-based cohort study by Lin et al. showed that patients with periodontitis have an approximately 50.0% increased risk of subsequent SD [27]. The meta-analysis by Wu et al. and the systematic review by Maarse et al. found no significant difference in PPD and CAL between pSD patients and control subjects [4,24]. However, the results of the above meta-analysis should be interpreted with caution due to the heterogeneity of the included studies [4]. The systematic review by de Góes Soares et al. found no clear evidence of the influence of SD on periodontitis [28]. Similar to the results of our cross-sectional study, Lugonja et al. and Yorkjend et al. failed to demonstrate that pSD patients have a significantly higher risk of developing periodontitis [29,30]. However, the high prevalence of periodontitis in pSD patients indicates the need to include a periodontist in the multidisciplinary team of SD. The detection and treatment of periodontitis is important because of its negative impact on OHRLoH, which is significantly impaired in pSD patients. This is confirmed by the study of Ambósio et al. which showed that the treatment of periodontitis in patients with pSD has beneficial effects and improves OHRQoL [31]. The oral medicine specialist plays a vital role in the multidisciplinary medical team involved in the diagnosis of SD, since the oral component is part of the classification criteria. In addition to the sialometry test, such an examination should also provide information on caries prevention, protection of teeth and soft oral tissues, methods to reduce dry mouth, and the importance of regular dental follow-up (Table 4). The results of our cross-sectional study showed that pSD has a negative impact on OHRQoL. Our results are consistent with the studies by Azum et al., Gobeljić et al., Rusthen et al., and the systematic review by Schmalz et al. showing significantly worse OHRQoL in SD patients compared to control subjects [32,33,34,35].

Objective (ESSDAI) and subjective (ESSPRI) indexes showed a strong statistically significant positive correlation with UWSFR and SWSFR. This suggests that whole saliva in pSD patients reflects disease activity, i.e., the identical pathophysiological mechanism of the disease. Unlike the subjective ESSPRI index, which showed a strong statistically significant positive correlation with UWSFR and SWSFR, this is not the case for the subjective indexes (6-items-VAS-SS, Profile of Fatigue), which showed a statistically significant negative correlation with the above oral cavity parameters. This can be explained by the fact that the subjective indexes (6-items-VAS-SS, Profile of Fatigue) cover six domains, while the ESSPRI index covers only three domains. This shows that other complications of pSD are more prominent (pain, somatic fatigue, arthralgia, myalgia, paresthesia, mental fatigue) and the oral cavity is unfairly neglected. This study clearly demonstrates the relationship between oral and systemic health, i.e., the importance of assessing the oral health of patients with pSD. Dry mouth and hyposalivation lead to decreased salivary buffering capacity, tooth demineralization, oral candidiasis, caries, gingivitis, periodontitis, and tooth loss. The results of our cross-sectional study can be used for targeted dental interventions to improve oral health and prevent systemic complications in patients with pSD. An oral medicine specialist and a periodontist should be part of the treatment algorithm for pSD. On the other hand, the objective (ESSDAI) and all subjective (ESSPRI, 6-items-VAS-SS, Profile of Fatigue) indexes showed a statistically significant negative correlation with pH. We can explain this by the fact that patients with pSD visit an oral medicine specialist more often, since the determination of UWSFR is one of the classification criteria for diagnosis. During the examination, patients with pSD receive oral hygiene instructions and dietary guidelines (Table 4), and their implementation leads to better oral hygiene and, consequently, oral pH within the reference limits. Interestingly, all indexes (ESSDAI, ESSPRI, 6-items-VAS-SS, Profile of Fatigue) showed a strong statistically significant positive correlation with the OHIP-49 total score. This suggests that both oral and systemic complications of pSD have a significant impact on reducing HRQoL in patients with pSD. The therapeutic approach for patients with pSD must be multidisciplinary and include both the physical and psychological aspects of the disease.

In our cross-sectional study, there was neither an association between periodontitis and the observed autoantibodies nor an increased risk of periodontitis in pSD patients. These results suggest that there is no bidirectional aetiology. Rheumatoid arthritis is an autoimmune disease most strongly associated with periodontitis. The systematic review by Wen et al. showed a significant association between the severity of clinical presentation of chronic periodontitis (CP) and the activity of RA [36]. The bidirectional relationship between periodontitis and RA is based on specific periodontal pathogens, such as *Porphyromonas gingivalis* and *Aggregatibacter actinomycetemcomitans*, involved in the citrullination process [37].

To the best of our knowledge, this is the first cross-sectional study of oral health in patients with pSD in Croatia. The limitations of our cross-sectional study include: being a single-center study, a small sample, lack of multivariate regression analysis, and the use of the Croatian version of the OHIP-49, which has not been validated for patients with pSD. We did not examine self-reported oral health attitudes and behaviors. The study by Vigu et al. showed that there is a complex interaction between oral health status, OHRQoL, and oral health-related attitudes and behaviors [38]. Attitudes and behaviors related to oral health in patients with pSD should be part of future studies with a larger number of subjects.

## 5. Conclusions

In conclusion, our cross-sectional study found decreased salivary flow and salivary pH, decreased interincisal distance, higher DMFT index, higher prevalence of periodontitis, and poorer OHRQoL in pSD patients compared to control subjects. Primary SD patients require a comprehensive therapeutic approach, and an oral medicine specialist and periodontist should be part of the SD multidisciplinary team caring for oral health.

## Figures and Tables

**Table 1:** Characteristics of pSD patients and control subjects.

	Sjögren’s Disease (n = 31)	Control Subjects (n = 31)	Z (*p*-Value)
	Mean (IQR) **	Mean (IQR) **	
Disease duration	5.50 (2.50–9.00)		
UWSFR (mL/min)	0.20 (0.00–3.00)	0.90 (4.00–5.50)	−5.74 (<0.001) *
SWSFR (mL/min)	0.56 (0.60–4.60)	1.64 (7.10–9.20)	−6.17 (<0.001) *
pH values	6.00 (5.50–6.50)	7.00 (6.40–7.00)	−3.77 (<0.001) *
ESSDAI	10.00 (7.00–14.00)		
ESSPRI	22.00 (15.00–25.00)		
6-items-VAS-SS	35.00 (28.00–42.00)		
Profile of Fatigue (VAS)	28.00 (22.00–36.00)		

* Mann–Whitney U test. ** Data expressed as mean and interquartile range (IQR). Abbreviations: ESSDAI, EULAR SS Disease Activity Index; ESSPRI, EULAR SS Patient Reported Index; SWSFR, stimulated whole salivary flow rate; UWSFR, unstimulated whole salivary flow rate.

**Table 2:** DMFT index and interincisal distance in pSD patients.

	Sjögren’s Disease (n = 31)	Control Subjects (n = 31)	t (*p*-Value)
	Mean (SD)	Mean (SD)	
Interincisal distance (mm)	43.80 (0.38)	47.60 (0.50)	−3.09 (0.003) *
DMFT index	23.74 (7.28)	20.77 (5.73)	1.78 (0.080) *

* *t*-test. Abbreviation: DMFT, D—decayed, M—missing, F—filled tooth.

**Table 3:** Correlation between observed variables with ESSDAI, ESSPRI, 6-items-VAS-SS, Profile of Fatigue.

		ESSDAI	ESSPRI	6-Items-VAS-SS	Profile of Fatigue
Disease duration	rho	0.277	0.185	0.262	0.315
	*p*	0.15	0.35	0.18	0.10
UWSFR (mL/min)	rho	0.708 **	0.650 **	−0.599 **	−0.630 **
	*p*	<0.001	<0.001	<0.001	<0.001
SWSFR (mL/min)	rho	0.743 **	0.703 **	−0.664 **	−0.700 **
	*p*	<0.001	<0.001	<0.001	<0.001
pH values	rho	−0.406 *	−0.441 *	−0.501 **	−0.413 *
	*p*	0.02	0.013	0.004	0.02
Total OHIP-49 score	rho	0.949 **	0.887 **	0.936 **	0.978 **
	*p*	<0.001	<0.001	<0.001	<0.001
Interincisal distance (mm)	rho	0.048	0.009	0.038	0.105
	*p*	0.83	0.97	0.86	0.62
DMFT index	rho	0.342	0.219	0.335	0.309
	*p*	0.06	0.24	0.07	0.09
Periodontitis	rho	0.017	0.074	0.034	0.017
	*p*	0.93	0.70	0.86	0.93

* *p* < 0.05, Spearman’s correlation. ** *p* < 0.01, Spearman’s correlation. Abbreviations: DMFT, D—decayed, M—missing, F—filled tooth; OHIP-49, Oral Health Impact Profile-49; SWSFR, stimulated whole salivary flow rate; UWSFR, unstimulated whole salivary flow rate.

**Table 4:** Oral hygiene instructions and dietary guidelines for SD patients.

The Salivary Glands Can Produce Saliva:	The Salivary Glands Cannot Produce Saliva:
(1) Local stimulation: - Moisten the oral cavity with small sips of liquid during the day (up to 1.50–2.00 L in total) - Occasionally drink slightly acidic beverages without sugar (e.g., lemonade) - Sugar-free chewing gum, three times a day for 30 min (2) Systematic stimulation recommended by specialists (anetoletrition, pilocarpine, cevimeline) (3) Therapeutic laser stimulation	- The use of artificial saliva in the form of solutions, sprays, and gels (without the addition of citric acid) - Moisten the oral cavity with small sips of liquid during the day (up to 1.50–2.00 L in total) - Drink liquid during meals to improve the taste and make it easier to swallow - Rinse the oral cavity with a warm solution of table salt and baking soda (1 teaspoon salt and 1 teaspoon baking soda to 2 dcl of water), every two hours to control salivary pH values
Oral hygiene instructions and dietary guidelines	
- Brush your teeth with a soft toothbrush after every meal, at least twice a day - Use interdental brushes, at least once a day - Use fluoride toothpaste - Use fluoride mouthwashes twice a day (should not contain alcohol or strong-tasting additives) * - Use fluoride gels, in the evening after brushing teeth, leave on for five minutes and do not rinse ** - Regular dental check-ups, at least four times a year - Moisten the oral cavity every 10 min during the day with small sips of liquid - Avoid sweet, very sour and spicy foods, solid and dry foods - Avoid alcohol, coffee, tea and smoking - Use humidifiers in winter (especially at night)	

* oral antiseptics should be diluted with water if they irritate the oral mucosa (1:1, 1:2, etc.). ** the dentist/oral medicine specialist determines the frequency of application based on the sialomtery test.

## Data Availability

Data are available at the corresponding author’s e-mail upon request.

## Appendix A

Modified self-assessment scale for subjective patient complaints 6-items-VAS-SS

1. **Dryness**      (0  1  2  3  4  5  6  7  8  9  10)
2. **Arthralgias**    (0  1  2  3  4  5  6  7  8  9  10)
3. **Myalgias**     (0  1  2  3  4  5  6  7  8  9  10)
4. **Paresthesias**     (0  1  2  3  4  5  6  7  8  9  10)
5. **Somatic fatigue**  (0  1  2  3  4  5  6  7  8  9  10)
6. **Mental fatigue**    (0  1  2  3  4  5  6  7  8  9  10)

*6-items-VAS-SS: minimum 0 to maximum 60
